# Evaluation of the solubility of 11-keto-β-boswellic acid and its histological effect on the diabetic mice liver using a novel technique

**DOI:** 10.14202/vetworld.2021.1797-1803

**Published:** 2021-07-12

**Authors:** Issa Al Amri, Fazal Mabood, Isam T. Kadim, Abdulaziz Alkindi, A. Al-Harrasi, Sulaiman Al-Hashmi, Ghulam Abbas, Ahmed Hamaed, Basant Ahmed, Jawaher Al-Shuhaimi, Samera Khalaf, Jamaluddin Shaikh

**Affiliations:** 1 Department of Biological Sciences and Chemistry, College of Arts and Sciences, University of Nizwa, PO Box 33, PC 616, Birkat Al-Mouz, Nizwa, Sultanate of Oman; 2 Institute of Chemical Sciences, University of Swat KP, Pakistan; 3 Natural and Medical Sciences Research Center, University of Nizwa, PO Box 33, PC 616, Birkat Al-Mouz, Nizwa, Sultanate of Oman; 4 School of Pharmacy, College of Pharmacy and Nursing, University of Nizwa, PO Box 33, PC 616, Birkat Al-Mouz, Nizwa, Sultanate of Oman

**Keywords:** 11-keto-β-boswellic acid, Fourier transform infrared reflectance, histology, liver, mice, principle component analysis

## Abstract

**Background and Aim:**

The literature is scant on the effect of 11-keto-β-boswellic acid (KBA) on the liver of diabetes-induced mice. This study was designed to develop a rapid, sensitive, accurate, and inexpensive detection technique for evaluating the solubility of KBA obtained from the gum resin of Omani frankincense (*Boswellia sacra*) in the liver of streptozotocin-induced diabetic mice using Fourier transform infrared (FTIR) reflectance spectroscopy coupled with principal components analysis (PCA). It also aimed to investigate the effect of KBA on histological changes in the hepatocytes of diabetic mice.

**Materials and Methods:**

Eighteen mice were assigned to the healthy control group, the diabetic control group, or the KBA-treated diabetic group. Liver tissue samples from all groups were scanned using an FTIR reflectance spectrophotometer in reflection mode. FTIR reflectance spectra were collected in the wavenumber range of 400-4000 cm^−1^ using an attenuated total reflectance apparatus.

**Results:**

FTIR reflectance spectra were analyzed using PCA. The PCA score plot, which is an exploratory multivariate data set, revealed complete segregation among the three groups’ liver samples based on changes in the variation of wavenumber position in the FTIR reflectance spectra, which indicated a clear effect of KBA solubility on treatments. Histological analysis showed an improvement in the liver tissues, with normal structures of hepatocytes exhibiting mild vacuolation in their cytoplasm.

**Conclusion:**

KBA improved the morphology of liver tissues in the diabetic mice and led to complete recovery of the damage observed in the diabetic control group. FTIR reflectance spectroscopy coupled with PCA could be deployed as a rapid, low-cost, and non-destructive detection method for evaluating treatment effects in diseased liver tissue based on the solubility of KBA.

## Introduction

Diabetes mellitus (DM) is a chronic metabolic disease characterized by hyperglycemia resulting from insufficient insulin action [1]. In 2020, the International Diabetes Federation (www.idf.org) reported that there were 463 million cases of diabetes worldwide, including 55 million in the Middle East and North Africa region and 291,000 in Oman. In 2017, 94,921 cases of diabetes were reported in Oman (according to the Ministry of Health 2017 Annual report, www.omanobserver.om). Moreover, the number of diabetes cases in the Middle East and North Africa region has been statistically estimated to increase to 108 million by 2045 [2].

DM causes several diseases, such as neurological disorders, coronary artery disease, renal failure, and cerebrovascular disease; limb amputation; blindness; as well as death [3,4]. Types 1 and 2 DM, gestational diabetes, impaired glucose tolerance, and impaired fasting glycemia are considered to be the main types of diabetes [1]. Type 1 DM is an autoimmune disorder, whereas type 2 DM is a metabolic disorder [5]. The human liver is the largest internal organ and has multiple functions, including regulation of the glucose concentration in physiological and pathological conditions as well as prevention of its excessive fluctuation [6]. Hyperglycemia affects the metabolism of protein, carbohydrates, and lipids, leading to nonalcoholic fatty liver disease (NAFLD) [7]. NAFLD can further progress to nonalcoholic steatohepatitis, cirrhosis, and, ultimately, hepatocellular carcinomas. Research has shown that NAFLD affects up to 70% to 80% of people with type 2 DM and up to 30% to 40% of people with type 1 DM [8].

Medicinal components extracted from the frankincense plant (*Boswellia* spp.) have been documented to possess health benefits and pharmaceutical properties, such as antimicrobial, anti-inflammatory, anticancer, antidiabetic, antioxidant, and other analgesic activities [9-14]. The active derivatives of the plant are the boswellic acids (BAs) found in the gum resin of *Boswellia* spp., which have pharmacologically active pentacyclic triterpene molecules, including 11-keto-b-BA (KBA) [10,13]. These active derivatives have been used to treat a number of inflammatory diseases, such as osteoarthritis, chronic colitis, bronchial asthma, pancreatic cells and tumors, human breast cancer cells [15], and hepatocellular carcinomas [16]. Frankincense has been reported to significantly increase the wound contraction rate after the administration of KBA for 16 days in diabetic mice [17]. In another study, diabetic mice injected with KBA for 7 weeks triggered the infiltration of a large number of lymphocytes into the pancreatic islets, and semi-isolated apoptotic cells were observed [18]. Research has also shown a decrease in the blood sugar level of diabetic mice after injection with KBA [19]. Pharmacokinetic studies, however, have evidenced low absorption of KBA in humans and rodents due to its poor water solubility and a strong tendency to self-aggregate [20-23]. One of such studies showed that the concentration of KBA declined with elimination of half-life after 6 h of oral administration and suggested that medication is required after every 6 h [23]. Therefore, strategies to improve the bioavailability of KBA need to be established to ensure its effective anti-inflammatory activity.

Fourier transform infrared (FTIR) reflectance spectroscopy is an effective tool used to determine the chemical characteristics of various sample forms, including solids, liquids, and gases. FTIR reflectance presents unique information by addressing cofactor, amino acid, and water molecule properties with high structural parameters and interactions [24]. Its advantages include rapid sample preparation and processing in solid forms for scanning with an FTIR reflectance spectrophotometer and subsequent analysis using a multivariate principal components analysis (PCA) method, which would allow for the development of a PCA model for exploring similarities and differences among samples based on the solubility of the drug compound.

To the best of our knowledge, the effect of intraperitoneal (IP) treatment with KBA on the liver of diabetes-induced mice has not been previously reported. This study analyzed the solubility of KBA extracted from the gum resin of Omani frankincense (*Boswellia sacra*) in the liver of streptozotocin (STZ)-induced diabetic mice and evaluated the effect of KBA on histological changes in the liver tissue under disease conditions using the IP route of administration.

## Materials and Methods

### Ethical approval

The use of the animals was approved by the Animal Ethics Committee at University of Nizwa.

### Study period and location

The study was conducted from September 2017 to July 2019, in DARIS Research Center, University of Nizwa, Oman.

### Experimental design and tissue sample preparation

Eighteen female CD-1 mice (weight, 25-30 g; age, 10-12 weeks) were used in this study. They were initially divided into two groups: The healthy control group (Group 1; n=6) and the STZ-induced group (Group 2; n=12). Six mice were kept in cages and exposed to a 12 h light/dark cycle, with controlled room temperature (24-25ºC) and humidity (30-35%.). All mice had access to food and water *ad libitum*. One week after adaptation, Group 1 mice were injected with citrate buffer, whereas Group 2 mice were given an IP injection of STZ (180 mg/kg body weight) in 10 mM citrate buffer (pH 4.5). Two weeks after the injection with STZ, blood samples were collected from the tail vein of the mice. STZ-induced mice with blood glucose levels >300 mg/dL were classified as diabetic mice and further divided into two experimental groups, each consisting of six mice: The diabetic control group and the KBA-treated diabetic group. The diabetic control mice (Group 2) received water only, whereas the diabetic experimental mice received IP injections of KBA dissolved in water at a dose of 25 mg/kg/d. Four weeks later, after the final treatment, the mice were anesthetized and killed. For light microscopy screening, liver tissues were dissected and immersed in 10% buffered formaldehyde solution; for FTIR reflectance analysis, liver tissues were frozen at −80°C in liquid nitrogen.

### FTIR reflectance spectroscopy

Each dried liver tissue sample was scanned using an FTIR reflectance spectrophotometer to produce reflectance spectra at five positions, with 16 scans per position to produce 80 spectra per sample. The averages of the 80 spectra per sample were used to build a multivariate PCA model.

### PCA

PCA is an unsupervised data exploration multivariate chemometric method used to show hidden similarities and differences among samples. In this study, Unscrambler X10.3 (CAMO Software, Oslo, Norway) and Microsoft Excel 2016 were used to create a PCA model of the FTIR reflectance spectral data. PCA was performed to determine the classification and segregation of the three groups’ dried liver tissue samples based on the solubility of KBA. The PCA model was built using the singular value decomposition algorithm. The internal validation of the PCA model was carried out using a leave-one-out, full cross-validation procedure, which involved investigating the maximum of seven principal components.

### Preprocessing of near-IR reflectance spectral data

Before the PCA modeling, unit vector normalization, standard normal variate transformation, and multiplicative scatter correction were employed to correct the multiplicative and additive effects of the spectra. Smoothing is usually applied to eliminate instrumental clutter or background information, and detrending methods are usually implemented to reduce the effects of accumulating data sets from a trend. The first and second derivatives of the spectra (D1 and D2) based on the Savitzky–Golay algorithm, with five smoothing points and polynomial order of 2, were also implemented to increase the spectral resolution. Derivatives are commonly used to reduce insignificant baseline signals from samples [25].

### Light microscopy

Tissue samples were fixed in 10% buffered formaldehyde solution for 8 h and then sliced into small pieces. The tissue slices were dehydrated in graded concentrations of ethyl alcohol, diaphonized in xylol, and then impregnated and embedded in paraffin wax. Sections (thickness, 4 μm) were produced by a rotary microtome and then stained with hematoxylin and eosin. For morphological examination, slides of stained sections were screened using a digital light microscope under 40×-1000×.

## Results

### FTIR reflectance analysis

**Figure-1:**
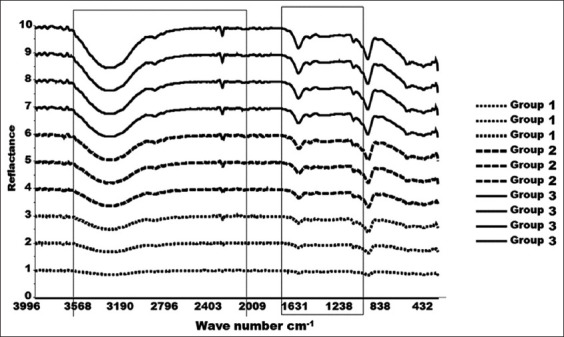
Fourier transform infrared spectra of solid liver tissue samples in reflectance mode for the three groups of mice. Group-1 normal liver tissue, Group-2 diabetic control liver tissue, and Group-3 treated intraperitoneal with 11-keto-β-boswellic acid tissue.

The FTIR reflectance spectra representing solid liver tissue samples from the three study groups were in reflection mode in the wavenumber range of 400-4000 cm^−1^ (Figure-1). They demonstrated a prominent increase in the peak intensities in the wavenumber range of 950-1650 cm^−1^ and in that of 2009-3568 cm^−1^ as KBA doses increased from Groups 1 to 3, which indicated a clear effect of KBA solubility on treatments. The FTIR reflectance spectra of pure KBA demonstrated characteristic peaks at 3437 cm^−1^ (O-H stretching), 2932 cm^−1^ (C-H stretching), 1697 cm^−1^ (C-O stretching of aryl acid), 1453 cm^−1^ (C-H bending), 1375 cm^−1^ (COO symmetric stretching of carboxylates), 1240 cm^−1^ (C-CO-C stretching of aryl ketone), as well as 1025 and 988 cm^−1^ (ring structures of cyclohexane) [26].

### PCA

**Figure-2:**
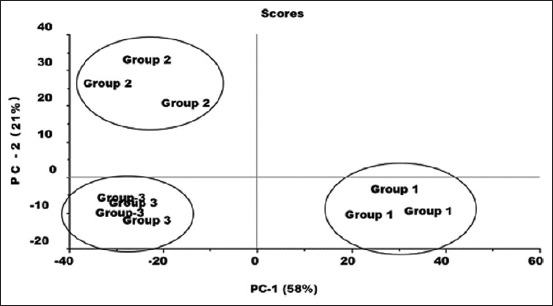
Principle component analysis score plot for three groups of mice liver samples (Group-1 normal liver, Group-2 diabetic control liver, and Group-3 diabetic liver treated intraperitoneal with 11-keto-β-boswellic acid).

PCA is an unsupervised multivariate exploratory data analysis tool that extracts such hidden information as similarities and differences based on variations in the data. Therefore, a PCA model (Figure-2) was built to extract masked information on the FTIR reflectance spectra for classification and discrimination among the three groups’ liver tissue samples based on KBA solubility. The PCA score plot (Figure-2) demonstrated a clear classification among the three groups based on the variation in FTIR reflectance peak intensities as well as peak position. The three groups’ tissue samples were mapped based on the solubility of KBA. Samples from Groups 1 and 3 were differentiated on the PC-1 axis, which utilized 58% of the spectral variation, whereas those from Groups 2 and 3 were differentiated on the PC-2 axis, which had 21% of the spectral information.

**Figure-3:**
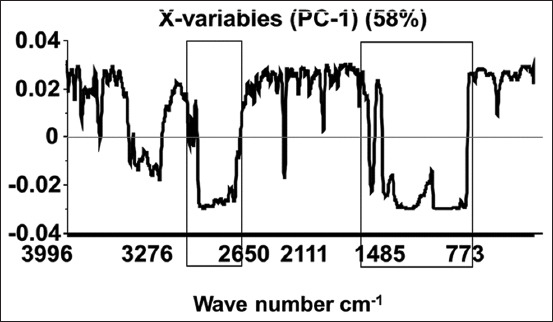
Principle component analysis loading plot.

PCA loadings were plotted to determine the part of the spectral variation that contributed to the PCA model (Figure-3). The loading plots for PC-1 demonstrated that the spectral regions in the wavenumber ranges of 950-1650 cm^−1^ and 2009-3568 cm^−1^ were the most important regions of the FTIR reflectance spectra and contributed mainly to the PCA model in increasing relevance from Groups 1 to 3, indicating a clear effect of KBA solubility on treatments – as KBA concentration peaks were distinctly elevated in Group 3.

### Histopathological analysis

**Figure-4:**
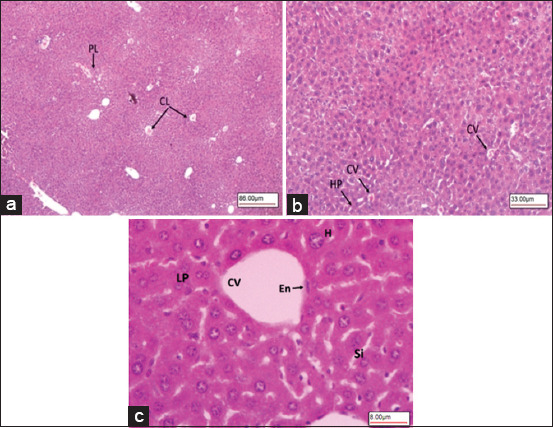
Light microscopy images of hematoxylin-eosin stained liver section showing normal control mice Group-1. (a) Liver histology made up of CL and PL. (b) CL consist of plates of HP radiating from a CV. (c) CV lined with En, H, LP, and liver Si (40×, 100×, and 400×, respectively). CL=Classical lobules, PL=Portal lobules, HP=Hepatocytes, CV=Central vein, En=Endothelium, H=Hepatocytes, LP=Liver hepatocyte plates, Si=Sinusoids.

Liver sections of the healthy mice treated with citrate buffer (Group 1) showed a liver architecture made of classical and portal lobules (Figure-4a). The classical lobules consisted of plates of hepatocytes radiating from a central vein and extending toward portal areas (Figure-4b). The portal lobules (triads) consisted of the hepatic artery, hepatic portal vein, bile duct, and lymphatic vessels (Figure-4). Between the hepatocyte plates were liver sinusoids, which are capillaries that carry blood from the hepatic portal vein, enter the hepatic artery through the portal triads, and then drain into the central vein (Figure-4b and c).

**Figure-5:**
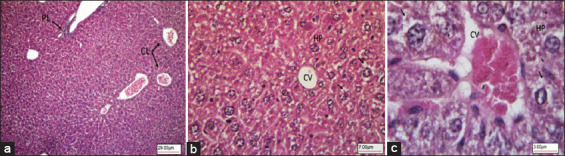
Diabetic control mice Group-2. (a) Liver tissue architecture. (b) Hepatocellular damage in the form of increased vacuolation in the cytoplasm of hepatocytes appeared as indistinct clear vacuoles (arrows) indicating possible glycogen infiltration and accumulation in diabetes. CV, HP. (c) Higher magnification image showing vacuoles in the cytoplasm of hepatocytes (arrows) (100×, 400×, and 1000×, respectively). CV=Central vein, HP=Hepatocytes.

**Figure-6:**
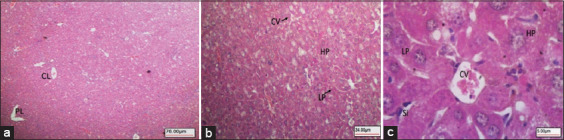
Diabetic mice IP treated with KBA Group-3. (a) Liver tissue appeared with normal architecture of CL and PL. (b) Normal HPs arranged in normal plates (LP) around CV. (c) High magnification showing normal HPs with mild vacuolation (HP) arranged in normal plates (LPs) around CV) Liver Si (40×, 400×, and 600×, respectively). IP=Intraperitoneal, KBA=11-Keto-β-boswellic acid, CL=Classical lobules, PL=Portal lobules, HPs=Hepatocytes, LP=Liver hepatocyte plates, CV=Central vein, Si=Sinusoids.

The histological results for the diabetic mice (Group 2) showed that the classical and portal lobules of liver tissues (Figure-5a) with histopathological changes, including hepatocellular damage in the form of increased vacuolation in the cytoplasm of hepatocytes, appeared as indistinct clear vacuoles, which indicated glycogen infiltration and accumulation (Figure-5b and c). The liver samples from the diabetic mice given IP injections of KBA (Group 3) showed a normal liver architecture (Figure-6a) with mild vacuolation in the cytoplasm of hepatocytes (Figure-6b and c).

## Discussion

DM is a metabolic disorder caused mainly by hyperglycemia and hyperlipidemia; it affects most of the body’s vital organs and involves multiple therapies for treatment [27]. Efficient treatments for certain types of diabetes (e.g., type 1 and late-onset autoimmune diabetes) are currently not available, but recent research has shown that the antidiabetic properties of BAs, the pharmacologically active constituents of frankincense, render them a viable therapeutic option [12]. The anti-inflammatory properties of BAs have been reported as targets for immune system factors involved in types 1 and 2 DM [28]. However, BA compounds, including α-BA and β-BA as well as acetylated α-BA and β-BA, were found to have no effect in inhibiting STZ-mediated hyperglycemia, whereas KBA and 3-*O*-acetyl-KBA were identified as key players in the prevention of diabetes. These isolated compounds, KBA and 3-*O*-acetyl-KBA, prevented the occurrence of autoimmune reactions as well as insulitis and reduced hyperglycemia in STZ-induced diabetes models [19]. Pharmacokinetic studies of *Boswellia* spp. extracts reported the poor bioavailability of these compounds in both humans and rats due to their poor water solubility and high lipophilicity [29,30]. Research has also shown that permeability-associated barriers that compromise the oral bioavailability of KBA include its gastrointestinal volatility, mediated intestinal metabolism, accumulation within the enterocytes, and saturable kinetics [31].

In this study, a rapid and inexpensive detection technique using FTIR reflectance spectroscopy coupled with a multivariate PCA method was developed to evaluate the solubility of KBA obtained from the gum resin of Omani frankincense in the liver of STZ-induced diabetic mice. This novel method demonstrated that the combination of FTIR reflectance spectroscopy and PCA was a reliable and accurate technique for the visualization of similarities and differences among the three groups’ mice liver tissue samples based on the solubility of KBA; it also showed a clear effect of KBA on treatments. The concentrations of KBA demonstrated distinct elevated peaks in the consolidated FTIR reflectance spectroscopy and PCA spectrum in the treated group. This result indicated that the concentration of KBA improved after IP treatment, which proved to be an effective route of administration, leading to increased KBA bioavailability. Several studies have used different approaches to enhance BA bioavailability [32,33], and many have reported the biologic effects of drugs after IP administration in animal models, suggesting the bioavailability of these large molecules as administered by said route [34,35]. Compared with the oral route of administration, the IP route has been proven to be more effective because it allows for a faster rate and a wider extent of drug absorption followed by the intramuscular route [36]. Pharmacological agents administered intraperitoneally are exposed to a large surface area, which leads to their rapid and efficient absorption [37].

The results of this study revealed that the high solubility of KBA in the animal model after IP treatment led to significant recovery of liver tissues and demonstrated the effect of KBA on histological alterations in the liver of STZ-induced diabetic mice. STZ is a naturally occurring compound that selectively destroys β-cells and induces alkylation of DNA, resulting in rapid necrosis, which leads to hyperglycemia [38]. Fresh liver tissue samples that were used for the solubility test were also used for histological examination. The histological findings for the diabetic mice showed classical and portal lobules in the liver tissues with histopathological changes, including hepatocellular damage in the form of increased vacuolation in the cytoplasm of hepatocytes appearing as indistinct clear vacuoles, which indicated glycogen infiltration and accumulation; these results are similar to previously reported findings [39]. After IP treatment with KBA, the liver tissues of diabetic mice markedly improved, with normal structures of hepatocytes exhibiting mild vacuolation in their cytoplasm. These results suggest that the active derivative of KBA from the gum resin of frankincense has a protective effect on the morphology of the liver of diabetic mice by significantly reducing hypoglycemia caused by STZ. Several studies have confirmed the effectiveness of KBA for diabetes and in suppressing the development of the disease in STZ-induced diabetic mice by triggering an immune response mediated by cytokines [12,19,40]. Furthermore, BAs have been reported to minimize liver complications and to provide a degree of protection against them [41]. Histological findings on liver tissues revealed that KBA has a protective effect against diabetes-induced damage [42]. The novel method herein described using FTIR reflectance spectroscopy coupled with PCA facilitated high segregation among the three groups’ mice liver tissues based on the solubility of KBA, which significantly improved after IP treatment, leading to significant recovery of the liver and hepatocytes.

## Conclusion

This study showed that FTIR reflectance spectroscopy coupled with PCA could be deployed as a rapid, inexpensive, and non-destructive detection method to examine the solubility of KBA in the liver of diabetic mice. BAs have been reported to effectively treat several inflammatory diseases, including diabetes. However, these active derivatives have low absorption in humans and rodents due to their poor water solubility. This limitation should be factored in when planning strategies to improve KBA solubility and its effectiveness in the treatment of DM. This study also showed that KBA has a curative potential as an antidiabetic and hepatic protective agent against STZ-induced diabetes damage by contributing to the regeneration of hepatocytes, thus providing supporting evidence for the high solubility of KBA in the animal model after IP treatment and the resulting significant recovery of liver tissues.

## Authors’ Contributions

IA, FM, GA, and SA: Conceived the experimental design. IA, GA, FM, SK, JA, and JS: Performed the experiment, data observation, and acquisition. IA, FM, ITK, AA, AbA, AH, SK, and BA: Analyzed and interpreted the data. IA, FM, AH, and ITK: Wrote the paper. All authors reviewed the manuscript, read, and approved the final manuscript.
